# Randomised Controlled Trials Assessing the Clinical Value of Urodynamic Studies: A Systematic Review and Meta-analysis

**DOI:** 10.1016/j.euros.2022.08.013

**Published:** 2022-09-05

**Authors:** Nicolas S. Bodmer, Carla Wirth, Veronika Birkhäuser, Andrea M. Sartori, Lorenz Leitner, Marcio A. Averbeck, Stefan de Wachter, Enrico Finazzi Agro, Andrew Gammie, Howard B. Goldman, Ruth Kirschner-Hermanns, Peter F.W.M. Rosier, Maurizio Serati, Eskinder Solomon, Gommert van Koeveringe, Lucas M. Bachmann, Thomas M. Kessler

**Affiliations:** a Department of Neuro-Urology, Balgrist University Hospital, University of Zürich, Zürich, Switzerland; b Medignition Inc., Research Consultants, Zürich, Switzerland; c Institute for Regenerative Medicine, University of Zürich, Zürich, Switzerland; d Department of Urology, Moinhos de Vento Hospital, Porto Alegre, Brazil; e Department of Urology, University Hospital Antwerp, University of Antwerp, Antwerp, Belgium; f Department of Surgical Sciences, University of Rome Tor Vergata, Rome, Italy; g Urology Unit, Policlinico Tor Vergata University Hospital, Rome, Italy; h Bristol Urological Institute, Southmead Hospital, Bristol, UK; i The Cleveland Clinic Foundation, Glickman Urological Institute, Cleveland, USA; j Neuro-Urology/Urology, University Clinic, Friedrich Wilhelms University Bonn, Bonn, Germany; k Johanniter Neurological Rehabilitation Centre, Bonn, Germany; l Department of Urology, University Medical Centre Utrecht, Utrecht, The Netherlands; m Department of Obstetrics and Gynecology, Urogynecology Unit, University of Insubria, Varese, Italy; n Urology Centre, Guy’s and St Thomas' NHS Trust, London, UK; o Department of Urology, Maastricht University Medical Centre, Maastricht, The Netherlands

**Keywords:** Urodynamics, Lower urinary tract symptoms, Urinary incontinence, Stress, Prostatic hyperplasia

## Abstract

**Context:**

The role of urodynamic studies (UDSs) in the diagnosis of lower urinary tract symptoms (LUTS) is crucial. Although expert statements and guidelines underline their value for clinical decision-making in various clinical settings, the academic debate as to their impact on patient outcomes continues.

**Objective:**

To summarise the evidence from all randomised controlled trials assessing the clinical usefulness of UDS in the management of LUTS.

**Evidence acquisition:**

For this systematic review, searches were performed without language restrictions in three electronic databases until November 18, 2020. The inclusion criteria were randomised controlled study design and allocation to receive UDS or not prior to any clinical management. Quality assessment was performed by two reviewers independently, using the Cochrane Collaboration’s tool for assessing the risk of bias. A random-effect meta-analysis was performed on the uniformly reported outcome parameters.

**Evidence synthesis:**

Eight trials were included, and all but two focused on women with pure or predominant stress urinary incontinence (SUI). A meta-analysis of six studies including 942 female patients was possible for treatment success, as defined by the authors (relative risk 1.00, 95% confidence interval: 0.93–1.07), indicating no difference in efficacy when managing women with UDS.

**Conclusions:**

Although UDSs are not replaceable in diagnostics, since there is no other equivalent method to find out exactly what the lower urinary tract problem is, there are little data supporting its impact on outcomes. Randomised controlled trials have focussed on a small group of women with uncomplicated SUI and showed no added value, but these findings cannot be extrapolated to the overall patient population with LUTS, warranting further well-designed trials.

**Patient summary:**

Despite urodynamics being the gold standard to assess lower urinary tract symptoms (LUTS), as it is the only method that can specify lower urinary tract dysfunction, more studies assessing the clinical usefulness of urodynamic studies (UDSs) in the management of LUTS are needed. UDS investigation is not increasing the probability of success in the treatment of stress urinary incontinence.

## Take Home Message

Despite urodynamics being the gold standard to assess lower urinary tract symptoms (LUTS), as it is the only method that can specify lower urinary tract dysfunction, more research assessing the clinical usefulness of urodynamic studies in the management of LUTS is critical.

## 1 Introduction

Over the past decades, invasive urodynamics (cystometry and pressure flow) have gained a pivotal role in the diagnostic work-up of lower urinary tract symptoms (LUTS) [1], [2] and are used to differentiate between different forms of urinary incontinence. However, despite various expert statements and some guidelines supporting the benefit of urodynamic investigations [3], [4], [5], their role in decision-making and patient management is still debated [6].

Led by an international group of specialised urologists, a comprehensive research programme was set out to evaluate the usefulness of urodynamics in various clinical domains. As a starting point, it was agreed that a paper should provide an inventory of the evidence across different fields of applications and different patient groups. This inventory should serve as a launching point and shortlist a series of systematic reviews focussing on specific clinical aspects or specific research settings [7].

The current systematic review of this series focussed on the evidence provided within randomised controlled studies assessing the clinical usefulness of urodynamics-guided management versus any kind of alternative work-up to improve patient outcomes. The aim was to provide an overview of the evidence available from randomised interventions, as these are considered to be most useful for assessing the efficacy of diagnostic tests [8], [9], [10].

## 2 Evidence acquisition

This systematic review was performed according to the Preferred Reporting Items for Systematic Reviews and Meta-analyses (PRISMA) statement. The protocol for the review is available on PROSPERO (CRD42019118464).

### 2.1 Evidence retrieval

The full details of how potentially eligible studies were identified have been published previously [7]. In brief, an information specialist affiliated with the library of the University of Zürich, Switzerland, performed comprehensive searches using an iterative approach. This approach included repeated testing of the search algorithm in terms of recall and precision of a set of relevant papers. Consecutive searches were performed without language restrictions in three electronic databases ([Pre-]Medline, EMBASE, and Cochrane Library) from inception until November 18, 2020. Electronic searches are available from the authors on request. Reference lists of included studies and reviews found in the searches were additionally checked.

### 2.2 Selection criteria and data extraction

Abstracts of all identified studies were reviewed by two authors independently. Studies reporting on randomised controlled trials were selected and reviewed in full text. We selected papers for inclusion if these assessed the efficacy of using urodynamics versus other clinical work-up in the management of therapeutic interventions. Quality assessment was performed using the Cochrane Collaboration’s tool for assessing the risk of bias [11]. Study selection and extraction were made in parallel by two researchers independently.

### 2.3 Statistical analysis

Across all studies, we selected the outcomes most consistently reported and used these results for the meta-analysis. We summarised all available group-averaged success data from each study. The meta-analysis was performed using a fixed- and a random-effect model (*metan* command in Stata; StataCorp LLC, College Station, TX, USA). If the results of both analyses were similar, we decided to report the results of the random-effect model. Analyses were performed using the Stata 16.1 software package (Stata Statistical Software: Release 16, 2019; StataCorp LLC).

## 3 Evidence synthesis

**Fig. 1:**
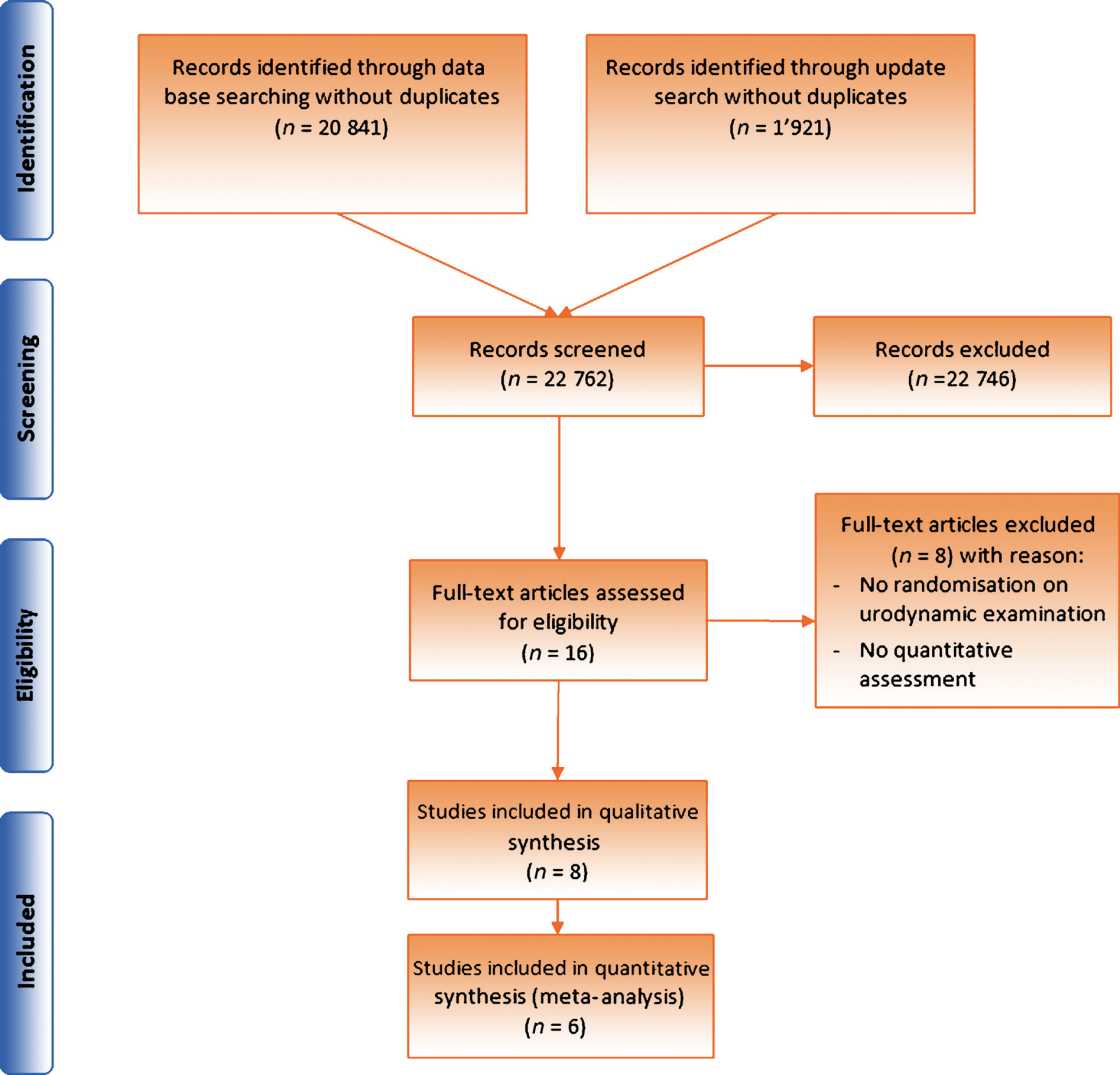
Preferred Reporting Items for Systematic Reviews and Meta-analyses flow diagram.

Figure 1 shows the PRISMA flow diagram of the literature search and results. Electronic searches identified 22 762 records and retrieved 16 randomised trials. After reading full texts, eight studies were excluded as these did not randomise for urodynamic examination or did not report a quantitative outcome. In total, eight randomised studies were included in the systematic review [12], [13], [14], [15], [16], [17], [18], [19]. With the exception of the studies by de Lima and Netto [18] and Drake et al. [19] that investigated men with bothersome LUTS considering prostate surgery, all other studies enrolled women with pure or predominant stress urinary incontinence (SUI). One study was only available in abstract form [15]. A summary of all included studies is provided in Table 1.

**Table 1:** Population, baseline characteristics

Author (year)	Recruitment start	Follow-up/duration (mo)	Number of centres	Noninferiority trial (Y/N)	Number of patients	Mean age (SD)	Mean BMI	Mean duration of symptoms (mo)	Previous surgery (eg, hysterectomy)	Postmenopausal
Agarwal (2014) [12]	Sept 2011	12	NR	N	Arm 1: 30 Arm 2: 30	Arm 1: 49.9 (11.4) Arm 2: 51.2 (12.1)	Arm 1: 26.4 Arm 2: 26.1	NR	Arm 1: 70.0% Arm 2: 76.7%	Arm 1: 43.3% Arm 2: 46.7%
de Lima (2003) [18]	Mar 1993	6	NR	N	Arm 1: 164 Arm 2: 151	Arm 1: 61 Arm 2: 63	NR	NR	NR	NA
Drake (2020) [19]	Oct 2014	18	26	Y	Arm 1: 427 Arm 2: 393	Arm 1: 67.5 (9.6) Arm 2: 67.8 (8.8)	NR	NR	NR	NA
Holtedahl (2000) [13]	1994	12	NR	N	Arm 1: 45 Arm 2: 42	Arm 1: 60.0 (6.3) Arm 2: 61.2 (7.5)	Arm 1: 26.8 Arm 2: 26.4	NR	NR	NR
Nager (2012) [14]	Nov 2008	12	11	Y	Arm 1: 264 Arm 2: 259	Arm 1: 51.9 (10.4) Arm 2: 51.6 (10.0)	Arm 1: 29.1 Arm 2: 28.9	Arm 1: 107.4 Arm 2: 90.7	Arm 1: 67.8% Arm 2: 74.1%	Arm 1: 45.1% Arm 2: 46.7%
Maroto (2010) [15]	Sept 2004	Arm 1: 46 Arm 2: 49	NR	N	Arm 1: 42 Arm 2: 44	NR a	NR a	NR	NR	NR a
van Leijsen (2012) [16]	Aug 2007	24	10	Y	Arm 1: 31 Arm 2: 28	Arm 1: 44 Arm 2: 43	Arm 1: 26 Arm 2: 24	NR	Arm 1: 20% Arm 2: 25%	Arm 1: 33% Arm 2: 19%
van Leijsen (2013) [17]	2009	24	30	Y	Arm 1: 62 Arm 2: 64	Arm 1: 54 (14) Arm 2: 55 (12)	Arm 1: 27 Arm 2: 27	NR	Arm 1: 27% Arm 2: 34%	NR

Arm 1 = urodynamic evaluation; Arm 2 = without urodynamic evaluation; Aug = August; BMI = body mass index; N = no; Mar = March; NA = not applicable; Nov = November; NR = not reported; SD = standard deviation; Sept = September; Oct = October; Y = yes.

a Age, BMI, parity, menopause, and number of pads were similar in both groups.

### 3.1 Inclusion criteria

In the 2012 study, van Leijsen and colleagues [16] investigated uncomplicated SUI—considered symptoms of pure SUI—or mixed urinary incontinence (MUI) with predominant SUI symptoms, who had previously failed conservative therapy and were candidates for surgical therapy. In the 2013 study, van Leijsen and coworkers [17] examined women if they had SUI or MUI with predominant SUI symptoms. To be included in the study, conservative therapy required to have failed and patients needed to be candidates for surgical treatment. Furthermore, incontinence suggestive of SUI required to have been demonstrated on physical examination and/or micturition diary.

Agarwal et al. [12] considered women presenting with predominant SUI (defined as involuntary leakage during physical activity, coughing, or sneezing). Holtedahl and colleagues [13] enrolled incontinent women reporting two or more leakage episodes per month according to the International Continence Society criteria. In the study of Nager and coworkers [14], women were eligible for inclusion if they were 21 yr of age or older, and had a history of symptoms of SUI for at least 3 mo, a positive stress test in the office, and uncomplicated SUI. Maroto et al. [15] enrolled women with SUI or MUI.

The study of de Lima and Netto [18] enrolled male patients with LUTS who were to have a transurethral resection of the prostate. In 2020, Drake et al. [19] published a large, multicentre, noninferiority, randomised study of men with bothersome LUTS, in whom surgery was an option. Participants were randomised to routine diagnostic work-up or an additional urodynamic examination. The primary outcome was noninferiority of 1 point in the International Prostate Symptom Score (IPSS; patient-reported outcome scale: from 0 to 35 points) 18 mo after randomisation between the two groups. Urological surgery rates were a key secondary outcome. The intervention arm showed noninferiority of the mean IPSSs at 18 mo (difference in IPSS of –0.33; 95% confidence interval [CI]: –1.47 to 0.80). In the urodynamic arm, 153/408 (38%) received surgery compared with 138/384 (36%) in the control arm (adjusted odds ratio 1.05; 95% CI: 0.77, 1.43) [19]. A detailed description of the inclusion criteria is available in Table 2.

**Table 2:** Inclusion/exclusion criteria and type of urodynamic studies

Author	Year	Inclusion criteria	Exclusion criteria	Type of urodynamics
Agarwal [12]	2014	Women presenting with predominant SUI (defined as involuntary leakage during physical activity, coughing, or sneezing) underwent a standardised basic office evaluation and were eligible for the study if they had a history of symptoms of uncomplicated SUI for at least 3 mo and failure to respond to standard medical treatment and pelvic floor exercises, postvoid residual of <150 ml, negative urine culture to exclude urinary tract infection, desire for surgery for SUI, and positive provocative stress test (defined as an observed transurethral loss of urine that was simultaneous with a cough or Valsalva manoeuvre). The provocative stress test, which had to be positive for inclusion in the study, was performed at the time of cystoscopic confirmation for SUI at a volume of approximately 300 ml along with a Bonney test. A clinical assessment of urethral mobility (defined as a straining angle of ≥30° relative to the horizontal on the Q-tip test) was also conducted at the same time.	Exclusion criteria were previous surgery for incontinence, history of pelvic irradiation, pelvic surgery within the previous 3 mo, and anterior or apical pelvic organ prolapse beyond 1 cm proximal to the hymen (stage II and higher of the pelvic organ prolapse quantification system). A random 150 ml cut-off for postvoid residual was chosen to exclude most patients with voiding dysfunction or neurogenic lower urinary tract dysfunction.	Standardised urodynamic testing included noninvasive uroflowmetry, filling cystometry with VLPP, urodynamic stress test, pressure flow studies, and urethral pressure profilometry. These were performed on the urodynamic group prior to surgery by the use of a multichannel urodynamics system (Dorado KT; Laborie, Toronto, ON, Canada). The International Continence Society–recommended Good Urodynamic Practice Guidelines were adhered to in the research. Conventional filling cystometry was performed with the patients in the supine position by using a 6 French double lumen catheter. The bladder was filled at a constant rate of 20 m/min by using normal saline solution at room temperature for standard urodynamic study. Simultaneous abdominal pressure monitoring was obtained through a fluid-filled rectal balloon catheter. Pressures were measured by using external pressure transducers that were zeroed to atmospheric pressure by using the level of the symphysis pubis as the reference height. The presence of involuntary detrusor contractions with or without incontinence was documented, and VLPP was obtained at a bladder volume of 200 ml. With the patient having a full bladder, the resting urethral pressure profile was obtained by using a 6 French catheter, catheter withdrawal, and water perfusion. The mean maximum urethral closure pressure of three successive withdrawals was used for a statistical analysis. Pressure flow study was subsequently performed with maximum cystometric capacity.
De Lima [18]	2003	Male patients with LUTS submitted to transurethral resection of the prostate were included.	Patients were excluded from the study if they had been exposed to drugs, such as alpha-agonists, anticholinergics, cholinergics, diuretic agents, oestrogens, androgens, antihypertensive medications, or other agents within the previous 2 wk. Other exclusion criteria consisted of a history or evidence of prostate cancer, pelvic irradiation, urethral stricture, or surgery for benign prostatic hyperplasia, or evidence of active urinary tract stone disease, neurogenic lower urinary tract dysfunction, hydronephrosis, or urinary tract infection within the 3 mo before the study.	Urodynamic evaluation was performed using the Urosystem/DS-5600 apparatus, connected to a 6 French rectal catheter for recording the abdominal pressure and a 6 French urethral catheter for recording the vesical pressure, with the patient standing. The catheters were connected to pressure transducers located at the level of the patient’s pubic symphysis. For the filling of the bladder, 0.9% saline infusion was introduced via an 8 French urethral catheter at an infusion rate of 50 ml/min. The vesical and abdominal pressures were recorded, as well as the detrusor pressure (defined as the vesical pressure minus the abdominal pressure) and uroflow rate. This examination included cystometry and pressure flow study, and in all patients, the measures were obtained in duplicate. The bladder outlet obstruction factor was defined in accordance with the criteria established by the International Continence Society. Utilising the Qmax and the PdetQmax, it was seen that: (1) when PdetQmax – 2 Qmax is >40, the pressure flow study indicates obstruction; (2) when PdetQmax – 2 Qmax is <20, the pressure flow study indicates absence of obstruction; and (3) in intermediate situations, the test result is equivocal obstruction.
Drake [19]	2020	Men (>18 yr old) with bothersome LUTS, in whom surgery was potentially being considered, were included.	The exclusion criteria were catheter use for bladder emptying, relevant neurological disease, current treatment for prostate or bladder cancer, previous prostate surgery, unfit for surgery, and/or unwillingness to comply with trial requirements.	Quality of urodynamic testing was according to the International Continence Society Good Urodynamic Practice requirements. It included the following: filling cystometry (detrusor overactivity and maximum cystometric capacity [ml]), pressure flow study (voided volume [ml], Qmax [ml/s], and postvoid residual [ml]), Bladder Contractility Index, and Bladder Outlet Obstruction Index. All 26 participating centres were checked for compliance with the international requirements beforehand.
Holtedahl [13]	2000	Inclusion criteria included fulfilling the International Continence Society criteria for urinary incontinence. Leakage was objectively demonstrated in at least one of three ways: visible leakage on coughing during the gynaecological examination, a positive 48 h pad test, or a recording of “wet” on a 48 h frequency-volume chart. The woman’s informed consent to participate in the treatment study was accepted as a confirmation that the patient experienced a social or hygienic problem, and only women reporting two or more leakage episodes per month were invited to join the study.		Urodynamic examination was performed by the two gynaecologist authors, one at Tromsø University Hospital and the other at Nordland Central Hospital in Bodø. The examination consisted of measurement of postvoid residual, filling of the bladder with 300 ml water at 37°C, urethral pressure profile, stress test by coughing, and subsequently 20 split jumps with pad weighing before and after, followed by cystometry, cystoflowmetry, cystoscopy (omitted on repeat examination), and gynaecological examination.
Nager [14]	2012	Women presenting with urinary incontinence underwent a standardised basic office evaluation and were eligible for the study if they were 21 yr of age or older, had a history of symptoms of SUI for at least 3 mo, and had a score on the Medical, Epidemiological, and Social Aspects of Aging questionnaire for SUI that was greater than the score on this questionnaire or urgency incontinence, postvoid residual of <150 ml, negative urinalysis or urine culture, clinical assessment of urethral mobility, desire for surgery for SUI, and positive provocative stress test (defined as an observed transurethral loss of urine simultaneous with a cough or Valsalva manoeuvre at any bladder volume).	Exclusion criteria were previous surgery for incontinence, history of pelvic irradiation, pelvic surgery within the previous 3 mo, and anterior or apical pelvic-organ prolapse of ≥1 cm distal to the hymen.	Women in the urodynamic-testing group underwent noninstrumented uroflowmetry with a comfortably full bladder, filling cystometry with VLPP, and a pressure-flow study. Urethral pressure profilometry or urodynamic testing with the use of video was permitted if it was performed routinely as part of the preoperative investigation at the study site. Testing followed the Good Urodynamic Practice guidelines of the International Continence Society, and interpretation conformed to International Continence Society nomenclature.
Maroto [15]	2010	Women with stress or mixed urinary incontinence were included	Patients younger than 18 yr of age and those who have had radiotherapy or any anti-incontinence procedure were excluded.	Urodynamics was used to determine maximum cystometric capacity (ml), detrusor pressure at Qmax (cmH_2_O), VLPP (cmH_2_O), and detrusor overactivity (%)
van Leijsen [16]	2012	Women were eligible for the study when they had SUI or MUI with predominant symptoms of SUI. To be included, conservative therapy must have failed, and patients were opting or candidates for surgical treatment. Furthermore, incontinence suggestive for SUI must have been demonstrated on physical examination and/or bladder diary.	Patients with previous incontinence surgery, pelvic organ prolapse >1 cm beyond the level of the hymen (POP-Q stage 3 or more), and/or postvoid residual of >150 ml on ultrasound or catheterisation were excluded.	Urodynamics investigation was performed according to the International Continence Society standards, and consisted of free uroflowmetry and postvoid residual measurement, filling cystometry with abdominal leak point pressure measurement, and pressure flow study. Urethral pressure profilometry in rest and during stress was optional. Eight centres used urodynamic equipment of Medical Measurement Systems, one centre used Andromeda equipment, and the remaining centre used equipment of Medtronic. The catheters (in two centres air charged; in eight centres water filled) had a size of 7 French. Standard filling speed was 50 ml/min; filling and voiding cystometry were performed with the patient in sitting position. For every 100 ml, a cough test was performed.
van Leijsen [17]	2013	Women with uncomplicated SUI considered as symptoms of pure SUI or MUI with predominant SUI symptoms, who had previously failed conservative therapy and were candidates for surgical therapy, were eligible for study inclusion. SUI was defined as self-reported complaints of involuntary loss of urine on effort, physical exertion, coughing, or sneezing. Women were considered to have predominant SUI in cases in which they reported the complaint of SUI and also involuntary loss of urine associated with urgency symptoms, and experienced the most bother of the stress component. SUI must have been demonstrated on physical examination or indicated on bladder diary, or both. A cough stress test was performed in the lithotonic position with a subjective full bladder. Postvoid residual was measured by catheterisation, ultrasonography, or bladder scan.	Patients were excluded if they had prior incontinence surgery or pelvic organ prolapse with the leading edge of prolapse at least 1 cm beyond the level of the hymen, or if a postvoid residual of ≥150 ml was present on ultrasonography or catheterisation.	All eligible women underwent urodynamics performed according to the International Continence Society standards. Free uroflowmetry was assessed using the Liverpool diagram. Measured parameters were Qmax (ml/s), postvoid residual (ml), maximum cystometric maximum (ml), and maximum urethral closure pressure (mmHg).

LUTS = lower urinary tract symptoms; MUI = mixed urinary incontinence; PdetQmax = detrusor pressure at the maximum flow rate; Qmax = maximum flow rate; SUI = stress urinary incontinence; VLPP = Valsalva leak point pressure.

### 3.2 Outcomes assessed

The studies were heterogeneous in respect to the number and definitions of various clinical outcomes.

Within the subgroup of studies on women qualifying for a meta-analysis, various measurement methods for SUI cure were available; a summary of the outcomes used in the meta-analysis is provided in Table 3. van Leijsen et al. [16], [17] used either “absence of SUI after 1 yr” or “total cure of SUI” as the combination of total subjective and total objective cure. Agarwal et al. [12] defined success as a reduction in the score on the Urogenital Distress Inventory of ≥70% at 12 mo after the onset of treatment, while Holtedahl et al. [13] defined improvement in the categories “cured”, “improved”, “unchanged”, and “worse”. The study by Nager and colleagues [14] defined success as a reduction in the score on the Urogenital Distress Inventory of ≥70% or more. Finally, Maroto et al. [15] defined improvement in the categories “dry”, “improved”, and “failure”.

**Table 3:** Results successful/unsuccessful treatment/classification

Author (year)	Urodynamic group: improvement (*n*)	Urodynamic group: no improvement (*n*)	Nonurodynamic group: improvement (*n*)	Nonurodynamic group: no improvement (*n*)	Definition of improvement
Agarwal (2014) [12]	27	3	20	10	Reduction in the Urogenital Distress Inventory (UDI-6) score of ≥70% at 12 mo after the onset of treatment
De Lima (2003) [18]	148	16	124	27	No obstruction after TURP surgery based only on urodynamics (urodynamic criteria listed in Table 2)^a^
Drake (2020) [19]	255	153	246	138	Improvement assessment = surgical rates within 18 mo of randomisation: Improvement = no surgery conducted No improvement = surgery conducted
Holtedahl (2000) [13]	25	19	24	17	Improvement = cured and improved No improvement = unchanged and worse
Nager (2012) [14]	210	62	210	56	Reduction in the UDI-6 score of ≥70%
Maroto (2010) [15]	41	1	43	1	Urinary incontinence grade cough test: improvement = dry + improved; no improvement = failure
van Leijsen (2012) [16]	17	14	20	8	Total cure of SUI was defined as the combination of total subjective and total objective cure. (Subjective cure of SUI was defined as a no leakage reported during physical activity [UDI]. Objective cure of SUI was defined as a negative stress test by physical examination.)
van Leijsen (2013) [17]	42	14	43	15	Absence of SUI after 1 yr (urodynamic = individually tailored treatment; nonurodynamic = surgery)

SUI = stress urinary incontinence; TURP = transurethral resection of the prostate; UDI = urodynamic investigation.

a No statistical significant difference regarding symptoms/uroflow.

### 3.3 Quality assessment

The studies available as full text were of mixed methodological quality. While randomisation was usually well performed, description of patient flow through the study, methods for analysing results (including an intention-to-treat analysis), handling of missing data, and other parameters revealed a relevant risk of bias. A complete summary of the quality assessment of each study is available in Table 4.

**Table 4:** Quality assessment items

Author (year)	Random sequence generation	Stratified randomisation	Allocation concealment	Blinding of patients	Blinding of therapists	Blinding of outcome assessors	Incomplete outcome data	Selective reporting	Baseline similarity of the arms	Patient flowchart reported	Intention-to-treat-analysis	Per-protocol analysis	Missing data	Dropouts/withdrawals, *n* (%)
Agarwal (2014) [12]	NR	NR	Unclear	Unclear	Yes	Unclear	Yes	No	Yes	Yes	Unclear	Unclear	Unclear	12 (17)
De Lima (2003) [18]	NR	NR	Unclear	Unclear	Unclear	Unclear	No	No	Unclear	Yes	Unclear	Unclear	Unclear	0 (0)
Drake (2020) [19]	Telephone/web-based system (computer-based randomisation)	No	Yes	No	No	No	No	No	Yes	Yes	Yes	Yes	No	151 (18)
Holtedahl (2000) [13]	Random number table	NR	Yes	No	No	Yes	Yes	Yes	Yes	No	Unclear	Unclear	Unclear	3 (4)
Nager (2012) [14]	Automated randomisation system	By surgeon	Yes	No	No	Yes	No	No	No^a^	Yes	Yes	Yes	Unclear	107 (17)
Maroto (2010) [15]	NR	NR	Unclear	Unclear	Unclear	Unclear	No	No	Yes	Unclear	Unclear	Unclear	Unclear	0 (0)
van Leijsen (2012) [16]	Computer-generated with remote computer access, blinded block sizes	By centre with a 1:1 allocation	Yes	No	No	No	No	No	Yes	Yes	Yes	No	LOCF	0 (0)
van Leijsen (2013) [17]	Web-based application, block randomisation, variable block size (2–8)	By centre with a 1:1 allocation	Yes	No	No	Yes	No	No	Yes	No	Yes	Yes	Unclear	11 (9)

LOCF = last observation carried forward; NR = not reported.

a Post hoc analysis with adjustment for baseline differences did not alter the findings.

### 3.4 Meta-analysis results

**Fig. 2:**
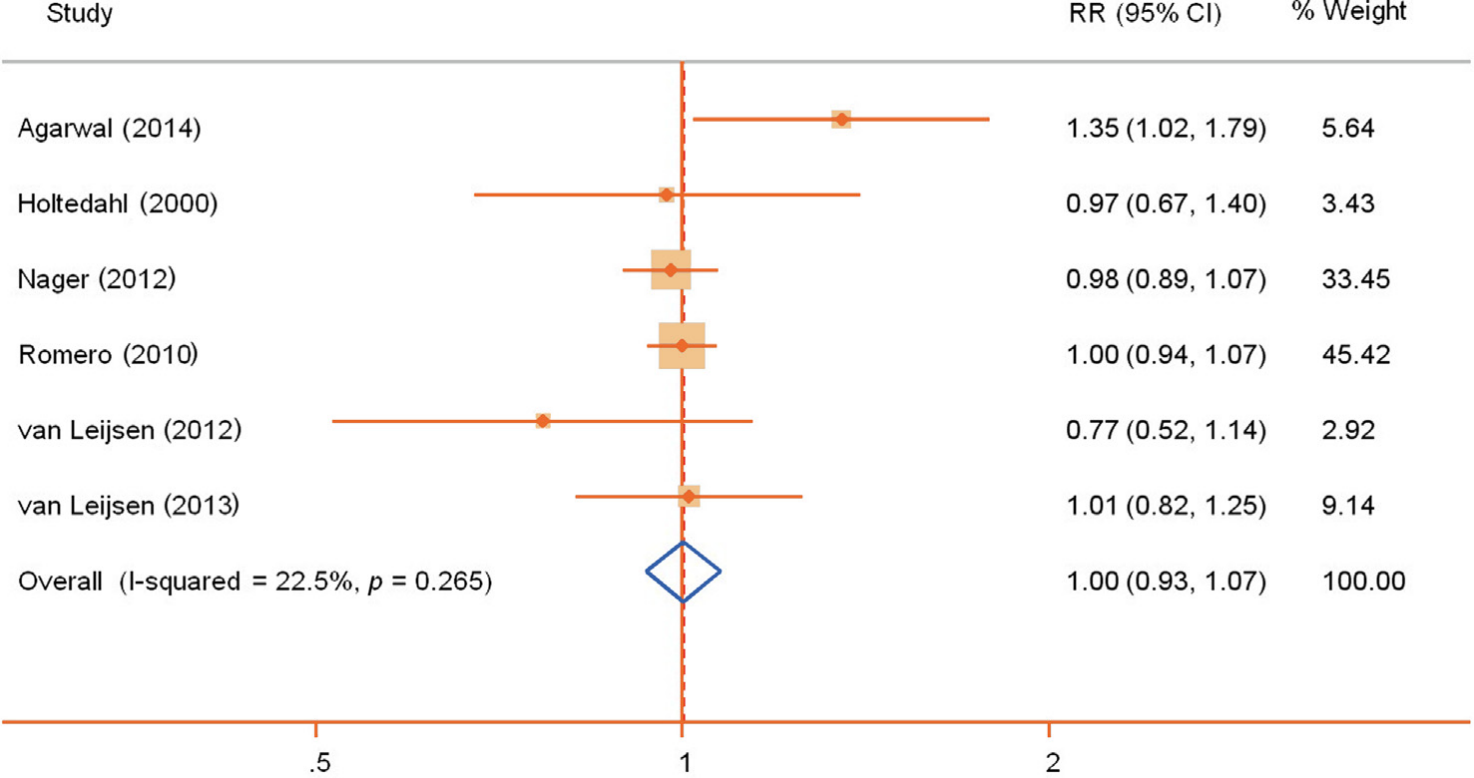
Meta-analysis (random-effect model) summarising the efficacy of urodynamic studies for treatment success. Treatment success is defined as symptom improvement as defined individually in the different studies. CI = confidence interval; RR = relative risk.

For the meta-analysis, six of the eight studies with a total of 942 patients could be included. Of all patients, 57% were sourced from the study by Nager et al. [14]. The 2 × 2 tables for the meta-analysis are shown in Table 3. The relative risk for treatment success outcomes using a random-effect model was 1.00 (95% CI: 0.93–1.07), indicating no difference in efficacy when managing patients with or without invasive urodynamics (Fig. 2).

## 4 Discussion

### 4.1 Main findings

All but two randomised controlled trials assessed the efficacy of invasive urodynamics in the management of LUTS in women with SUI. The number of patients included in the different trials ranged from 59 to 523, and the largest single trial contributed more than half of all patients [14]. The primary outcome parameters to quantify a treatment success, the urodynamic protocols and the clinical assessments, varied substantially between the different studies. A meta-analysis of six trials of limited methodological rigour (Cochrane grading; moderate to low), including almost 1000 patients, showed no benefit of urodynamic testing in women suffering from SUI or MUI with predominant SUI symptoms in terms of an increased treatment success of surgical management. We remain, however, limited for most patients by a lack of evidence since the findings of the present study derived from a small subgroup cannot be extrapolated to the majority of patients with LUTS.

### 4.2 Results in context

Only a few randomised controlled trials that had the aim to assess urodynamic diagnosis could be included. Besides the two studies investigating men with bothersome LUTS considering prostate surgery [18], [19], all remaining trials studied women with SUI or MUI with predominant SUI symptoms, with diagnoses primarily made by medical history and noninvasive clinical examination (eg, pad test and stress test). Moreover, the female populations included in these randomised controlled trials were highly selected (predominately women with uncomplicated SUI), representing only a small proportion of all women with urinary incontinence [4]. In addition, most trials excluded patients with other concomitant storage symptoms (eg, predominant urgency) or voiding symptoms. Therefore, generalisability of the findings is very limited.

Invasive urodynamics is the gold standard to objectively assess dysfunction of the lower urinary tract. However, some patients can be treated without urodynamics, and understandably, there is a clinical trade-off between the risks of empiric management and the potential drawbacks of the preceding diagnostic activity. The more invasive or risky a treatment is, the more precise the diagnosis should likely be. On the contrary, even though not invasive, plaster cast management of a fracture is rarely done without a diagnostic confirmation by radiography [20]. Accepted reasons for this are documentation of the baseline situation, assessment of complexity, and/or selection of other management types when necessary. This could be the case for lower urinary tract dysfunction. Nevertheless, invasive minor surgery (ie, suburethral tapes) for SUI, particularly in uncomplicated cases with demonstrable urine loss with cough test and a normal urinary volume and frequency on bladder diary, is successful in around 60–70% of cases. From a recent paper by Nager and colleagues [21], we can assume that patients suffering from SUI, with Valsalva leak point pressure or maximum urethral closure pressure values in the lowest quartile of the distribution, are twice as likely to experience sling failure after 12 mo of observation. Guidelines will continue to support performing this surgery, based on clinical assessment if there is no professional (or societal) need to improve this. Moreover, at present, the evidence that urodynamics can improve outcomes in this very specific cohort is lacking. However, there is a consensus among most clinical experts that for patients with mixed symptoms and/or frequent voiding and urinary incontinence, urodynamics is of value, once lifestyle changes and medical management have failed to reduce symptoms. In addition, for patients with frequent voiding and nocturia without urinary incontinence, urodynamics can rule out detrusor overactivity as the cause of frequent voiding, thus preventing costly and invasive management. Experts also agree and guidelines recommend that patients who have failed lower urinary tract surgery and patients with neurogenic lower urinary tract dysfunction should undergo urodynamics. Importantly, for the pathophysiological understanding of LUTS, urodynamic investigation is not replaceable, since there is no other equivalent method to find out exactly what the problem is. Finally, if a woman has previously undergone midurethral sling surgery and presents with de novo overactive bladder symptoms, urodynamics can be valuable to diagnose bladder outlet obstruction, which may be the proximate cause of the overactive bladder symptoms.

The UPSTREAM study [19], which is a pragmatic, multicentre, two-arm (unblinded) randomised controlled trial carried out in the UK, showed that urodynamics before surgery for LUTS secondary to benign prostatic enlargement resulted in a symptom outcome that is noninferior to a routine care pathway. In addition, urodynamics did not affect surgery rates for treating bladder outlet obstruction. However, secondary publications are still awaited to define whether urodynamics may provide relevant prognostic information for selected subgroups of male patients undergoing surgery for benign prostatic enlargement.

### 4.3 Outlook and further research

A urodynamic investigation can be seen as a structured and standardised “stress” test for the lower urinary tract. This test interpreted in combination with the patient history, physical examination, and other assessments such as bladder diary, free uroflowmetry and postvoid residual measurement provides essential information in the diagnostic work-up. With a view where urodynamic examinations might have the greatest impact in clinical management, research in this field should particularly focus on complex patients in whom diagnosis is unclear. Moreover, these studies should examine and quantify the role of urodynamics to select treatment strategies and follow up patients in a more individualised manner. Testing of patients should have a defined role that proves clinically and economically worthwhile.

While searches retrieved a plethora of studies on urodynamics, there remains a paucity of randomised studies that actually measure the impact of urodynamics on outcomes in relevant patient groups. There are ample avenues for research into this area that could improve the quality of care received by patients. Further research is also needed to determine whether urodynamic studies are helpful in specific situations, such as in patients presenting with mixed LUTS and risk factors for progression of bladder outlet obstruction. Moreover, urodynamically comparing desobstruction effects of different methods, such as prostatic urethral lift, aquablation, prostatic artery embolisation, and laser surgery, versus transurethral resection of the prostate would be relevant. Finally, a randomised study evaluating whether urodynamics influences the outcomes of third-line overactive bladder therapy should be planned.

The role of urodynamics in female incontinence is still a hotly debated topic. Despite the findings of the VaLUE and VUSIS-II randomised clinical trials published in 2012, which suggested that urodynamics is not useful in women with uncomplicated SUI, several experts raised concerns as to the inappropriate generalisation of the findings beyond uncomplicated cases, which represent only a minority of the overall patient population [22]. For patients with MUI and pelvic organ prolapse, two clinically challenging and highly prevalent conditions in postmenopausal women, further studies could help define whether urodynamics may modify the management (by identifying the predominant component of urinary incontinence) and/or provide valuable prognostic information (the probability of success given a minimally invasive procedure).

### 4.4 Strength and limitations

This systematic review and meta-analysis was conducted according to the current recommendations. The comprehensive summary of the available trial evidence is a strength of this paper. Weaknesses are due to the relevance, number, and quality of the available studies and thus the relative lack of applicable evidence. We were able to provide a summary result for studies examining urodynamics with treatment outcome in females with uncomplicated SUI, but the analysis was limited to one (subjectively reported) outcome that was more or less comparable across the different reports. Moreover, the meta-analysis summarised the findings of <1000 patients and was heavily influenced by one study that contributed more than one-half of the patients and that was clearly limited by methodological issues (imbalance of the two study groups regarding relevant baseline characteristics such as oestrogen replacement therapy, urethral mobility, duration of incontinence, and Incontinence Severity Index score). Owing to the small number of studies, no subgroup analysis (meta-regression analysis) was possible.

## 5 Conclusions

Urodynamics is the only method that can specify lower urinary tract dysfunction, and it is undisputed as the gold standard to assess LUTS in many patients. On the contrary, clinical experience also teaches us that successful management of women with SUI without other LUTS is possible without urodynamic testing. Six randomised controlled trials assessing the efficacy of urodynamics-guided management of patients with SUI showed no added value for preoperative urodynamics in this specific group of patients. For most patients, however, we still have a lack of evidence as the results from randomised trials conducted in a small subgroup cannot be extrapolated to all relevant patients with LUTS. The current evidence is figuratively reminiscent of the description of a known uninhabited island rather than the exploration of the entire atoll.

  ***Author contributions*:** Lucas M. Bachmann had full access to all the data in the study and takes responsibility for the integrity of the data and the accuracy of the data analysis.

*Study concept and design*: Bachmann, Kessler.

*Acquisition of data*: Bodmer, Wirth, Birkhäuser, Sartori.

*Analysis and interpretation of data*: Bodmer, Bachmann, Kessler.

*Drafting of the manuscript*: Bodmer, Bachmann, Kessler.

*Critical revision of the manuscript for important intellectual content*: Wirth, Birkhäuser, Sartori, Leitner, Averbeck, de Wachter, Finazzi Agro, Gammie, Goldman, Kirschner-Hermanns, Rosier, Serati, Solomon, van Koeveringe.

*Statistical analysis*: Bodmer, Bachmann.

*Obtaining funding*: Bachmann.

*Administrative, technical, or material support*: Bachmann, Kessler.

*Supervision*: Bachmann, Kessler.

*Other*: None.

  ***Financial disclosures:*** Lucas M. Bachmann certifies that all conflicts of interest, including specific financial interests and relationships and affiliations relevant to the subject matter or materials discussed in the manuscript (eg, employment/affiliation, grants or funding, consultancies, honoraria, stock ownership or options, expert testimony, royalties, or patents filed, received, or pending), are the following: None.

  ***Funding/Support and role of the sponsor:*** The work performed in this study was funded via an unrestricted research grant of the Laborie-owned company Medical Measurement Systems, Enschede, The Netherlands. This funding source had no role in the design of this study and did not have any role during its execution, analyses, interpretation of the data, or decision to submit results.
